# Early postoperative hyponatremia after pituitary adenoma surgery: risk factors, predictive model, and clinical implications

**DOI:** 10.1186/s12902-026-02212-2

**Published:** 2026-03-03

**Authors:** Chunmei Yin, Fei Xu, Xian Jiang, Yunji Wang, Tingping Xie, Xiaoyan Wang, Yingying Deng, Yi Liu

**Affiliations:** 1 Department of Neurosurgery, Nanfang Hospital, Southern Medical University, Guangzhou, Guangdong China; 2 Anesthesia and Operation Center, Nanfang Hospital, Southern Medical University, Guangzhou, Guangdong China

**Keywords:** Pituitary adenoma, Early postoperative hyponatremia, Risk factors, Predictive model, Perioperative management

## Abstract

**Objective:**

Early postoperative hyponatremia (EPH, days 1–3) following pituitary neuroendocrine tumor (PitNET) surgery is a clinically significant complication with poorly defined risk factors. This study was designed to: (1) identify independent risk factors for EPH across all PitNET subtypes; (2) construct and validate a reliable predictive model using clinicopathological variables; and (3) develop a simplified risk scoring system to guide risk-stratified perioperative management.

**Methods:**

This retrospective cohort study analyzed data from 300 adult patients who underwent PitNET surgery (January 2021 – December 2024) at Nanfang Hospital. Demographics, tumor characteristics (size, Knosp grade, functional type), preoperative conditions, and surgical details were collected. EPH was defined as serum sodium < 135 mmol/L within 1–3 days postoperatively. Multivariate binary logistic regression with stepwise selection (entry criterion *P* < 0.10, removal criterion *P* > 0.10) was used to identify independent risk factors and construct the predictive model. Model performance was evaluated by the area under the receiver operating characteristic curve (AUC), calibration plot, Hosmer-Lemeshow test, and bootstrap resampling (1000 iterations) for internal validation. The optimal risk score cutoff was determined by the Youden index. This study followed the STROBE guidelines.

**Results:**

EPH occurred in 70 patients (23.3%). Five independent risk factors were identified: tumor diameter > 25 mm (OR = 2.85, 95% CI: 1.52–5.34, *P* = 0.001), Knosp grade ≥ 3 (OR = 3.10, 95% CI: 1.65–5.83, *P* < 0.001), adrenocorticotropic hormone (ACTH)-secreting tumor (OR = 3.55, 95% CI: 1.41–8.94, *P* = 0.007), preoperative hyponatremia (OR = 4.20, 95% CI: 1.81–9.74, *P* < 0.001), and operative duration > 180 min (OR = 2.15, 95% CI: 1.13–4.08, *P* = 0.019). The predictive model exhibited excellent discriminative ability (AUC = 0.85, 95% CI: 0.75–0.87) and adequate calibration (Hosmer-Lemeshow test: χ²=8.56, df = 8, *P* = 0.38), with robust performance confirmed by bootstrap validation (AUC = 0.83). A simplified risk scoring system (0–6 points) stratified patients into low- (0–1 points, EPH incidence 5.0%), medium- (2–3 points, 35.0%), and high-risk (≥ 4 points, 82.5%) groups. The optimal cutoff value (3 points) yielded a sensitivity of 78.6% and specificity of 83.9%.

**Conclusion:**

Tumor size (> 25 mm), invasiveness (Knosp grade ≥ 3), ACTH-secreting subtype, preoperative hyponatremia, and prolonged operative duration (> 180 min) are independent predictors of EPH. To our knowledge, this is the first comprehensive predictive model and simplified scoring system for EPH across all PitNET subtypes. The validated tool enables accurate preoperative risk stratification, facilitating personalized perioperative monitoring and targeted interventions to reduce EPH-related complications and improve patient outcomes.

**Supplementary Information:**

The online version contains supplementary material available at 10.1186/s12902-026-02212-2.

## Introduction

Pituitary neuroendocrine tumors (PitNETs), previously termed pituitary adenomas, account for 10–15% of all intracranial tumors [1, 2]. Endoscopic transsphenoidal surgery (TSS) is the first-line treatment for symptomatic lesions, aiming to achieve maximal tumor resection, alleviate mass effects, and restore endocrine function [1]. Despite advances in surgical techniques, postoperative complications remain a major concern, with electrolyte disturbances being among the most prevalent and life-threatening [3, 4].

Hyponatremia (serum sodium < 135 mmol/L) is a leading electrolyte abnormality following PitNET surgery, with severe cases associated with cerebral edema, seizures, altered consciousness, and death [5, 6]. Most existing studies have focused on delayed postoperative hyponatremia (DPH), which typically occurs 4–10 days postoperatively and is predominantly attributed to the Syndrome of Inappropriate Antidiuresis(SIAD) [7, 8]. In contrast, early postoperative hyponatremia (EPH)—defined as onset within 1–3 days postoperatively—has received less attention despite its clinical relevance. EPH exhibits distinct pathophysiological features from DPH: patients often present with normal or increased urine output, suggesting mechanisms other than classic SIAD. Postoperative sodium fluctuations often follow characteristic temporal patterns, including the classic triphasic response (polyuria, hyponatremia, and permanent diabetes insipidus) or diphasic patterns of antidiuretic hormone (ADH) dysregulation [3]. In contrast, EPH exhibits distinct pathophysiological features—including cerebral salt wasting syndrome (CSWS), acute hormone fluctuations (e.g., cortisol withdrawal), and postoperative inflammatory responses [9]. This mechanistic complexity makes EPH challenging to predict and manage, highlighting the need for targeted research.

A recent study on DPH in nonfunctioning PitNETs (NF-PitNETs) identified intraoperative cerebrospinal fluid (CSF) leak, preoperative thyrotropin (TSH) elevation, and lower postoperative day 1 (POD 1) serum sodium as key risk factors [8]. Another recent investigation focusing on EPH in NF-PitNETs reported a 25.6% incidence, with independent predictors including thiazide diuretic use, pituitary apoplexy, elevated preoperative glucose, and lower BMI (BMI > 27 as a protective factor) [10]. Notably, this study confirmed that EPH predicts subsequent DPH, emphasizing the clinical significance of early risk identification [10]. However, these studies either focus solely on NF-PitNETs or lack a practical risk stratification tool, limiting their generalizability to the broader PitNET patient population.

To address this gap, we conducted a retrospective cohort study to identify independent risk factors for EPH across all PitNET subtypes, develop a clinically applicable predictive model, and compare its characteristics with existing EPH and DPH studies. To our knowledge, this is the first study to develop a comprehensive predictive model covering all PitNET subtypes, addressing the critical gap of subtype-specific limitations in previous EPH research [10].

## Materials and methods

### Study design and participants

This single-center retrospective cohort study included adult patients (≥ 18 years) who underwent PitNET surgery (transsphenoidal or transcranial approach) at the Department of Neurosurgery, Nanfang Hospital, Southern Medical University, between January 2021 and December 2024. Clinical trial number: not applicable.

The diagnosis of PitNET was confirmed by postoperative pathology, in accordance with the 2022 WHO classification [1, 2]. Exclusion criteria were: (1) severe preoperative cardiac, hepatic, or renal insufficiency; (2) long-term preoperative use of medications affecting sodium metabolism (e.g., diuretics, carbamazepine); (3) missing critical perioperative data (e.g., serum sodium levels on days 1–3 postoperatively); (4) other sellar lesions (e.g., craniopharyngioma, meningioma).

Ethical Considerations: The studies involving humans were approved by the Ethics Committee of Nanfang Hospital. The studies were conducted in accordance with the Declaration of Helsinki. The requirement for informed consent was waived by the Ethics Committee of Nanfang Hospital because this study was a retrospective analysis of de-identified clinical data collected as part of routine patient care.

### Data collection

Data were extracted from the hospital’s electronic medical record system, including:

#### Demographics: age, sex

Tumor characteristics: Maximum tumor diameter (mm) measured by preoperative contrast-enhanced MRI; Knosp grade (0–4) assessed on preoperative contrast-enhanced MRI (coronal view); functional type classified by preoperative hormone profiles, clinical manifestations, and immunohistochemistry (nonfunctioning PitNET [NF-PitNET], growth hormone [GH]-secreting PitNET, prolactin [PRL]-secreting PitNET, adrenocorticotropic hormone [ACTH]-secreting PitNET (defined as patients with clinical Cushing’s disease and biochemical hypercortisolism; silent ACTH adenomas were classified as NF-PitNETs), thyroid-stimulating hormone [TSH]-secreting PitNET, gonadotropin [FSH/LH]-secreting PitNET);

Preoperative conditions: Serum sodium level after admission; pituitary insufficiency (deficiency in at least one axis: thyroid, adrenal, or gonadal; all patients received appropriate hormone replacement and were euthyroid/eucortisol at surgery); tumor apoplexy on preoperative MRI; recurrent surgery history; preoperative fasting time (hours);

Surgical factors: Surgical approach (transsphenoidal/transcranial); operative duration (minutes);

Outcome variable: EPH was defined as the lowest serum sodium concentration < 135 mmol/L on any of POD 1–3.

#### Perioperative management

Standardized perioperative management included routine stress-dose hydrocortisone replacement (typically 100 mg intravenously during induction, tapered over 3–5 days) for all patients to prevent perioperative adrenal crisis. Although early postoperative serum cortisol levels (e.g., POD 1) were measured clinically, these data were excluded from the statistical analysis. Since hydrocortisone is chemically identical to cortisol in laboratory assays, exogenous administration renders the assessment of endogenous hypothalamic-pituitary-adrenal (HPA) axis function uninterpretable.Postoperatively, patients were allowed free access to water unless SIAD was confirmed. Desmopressin was administered only if central diabetes insipidus (urine output > 300 mL/h for 2 consecutive hours, specific gravity < 1.005) was confirmed.

### Statistical analysis

Statistical analyses were performed using SPSS version 26.0 (IBM Corp., Armonk, NY, USA) and R software version 4.3.1 (The R Foundation for Statistical Computing, Vienna, Austria). Continuous variables were expressed as mean ± standard deviation (Mean ± SD) and compared using t-tests or Mann-Whitney U tests. Categorical variables were presented as counts and percentages and compared using chi-square tests or Fisher’s exact tests. Variables with *P* < 0.10 in univariate analysis were included in multivariate binary logistic regression with stepwise selection (entry criterion *P* < 0.10, removal criterion *P* > 0.10) to identify independent risk factors for EPH (*P* < 0.05), with odds ratios (ORs) and 95% confidence intervals (CIs) calculated. Continuous variables (tumor diameter and operative duration) were converted into binary variables based on optimal cutoff values determined by Receiver Operating Characteristic (ROC) curve analysis (Youden index) to maximize predictive sensitivity and specificity. The predictive model construction, calibration plots, receiver operating characteristic (ROC) curve analysis, and internal bootstrap validation were conducted using the ‘rms’ and ‘pROC’ packages in R software.

### Risk prediction model development and validation

Independent risk factors from multivariate analysis were incorporated into a logistic regression equation. A simplified risk scoring system was created by assigning integer scores based on OR values to facilitate clinical application. Model discrimination was evaluated by the AUC and its 95% CI. Calibration was assessed using a calibration plot and Hosmer-Lemeshow test. Internal validation was performed using bootstrap resampling (1000 iterations) to evaluate the robustness of the model. The optimal cutoff value of the risk score was determined by the Youden index (sensitivity + specificity − 1), with corresponding sensitivity and specificity reported.

## Results

### Baseline patient characteristics

A total of 300 patients were enrolled, with a mean age of 48.5 ± 12.1 years and 160 (53.3%) females. The mean maximum tumor diameter was 24.8 ± 11.5 mm, and 85 (28.3%) patients had Knosp grade ≥ 3. NF-PitNET was the most common subtype (155 cases, 51.7%), followed by GH-secreting PitNET (60 cases, 20.0%), PRL-secreting PitNET (45 cases, 15.0%), and ACTH-secreting PitNET (25 cases, 8.3%). Preoperative pituitary insufficiency was present in 130 (43.3%) patients, preoperative hyponatremia in 28 (9.3%), tumor apoplexy in 20 (6.7%), and recurrent surgery in 40 (13.3%). Most patients (275, 91.7%) underwent transsphenoidal surgery, with a mean operative duration of 165 ± 55 min (Table 1).Among the 28 patients with preoperative hyponatremia, 22 (78.6%) achieved normalization of serum sodium levels (> 135 mmol/L) within the first 3 days postoperatively following tumor resection.

**Table 1:** Baseline characteristics of patients with pituitary neuroendocrine tumors (PitNETs) (*n* = 300)

Feature	Value (*n* (%)) or Mean ± SD
Age (years)	48.5 ± 12.1
Sex (Female)	160 (53.3%)
Max. Tumor Diameter (mm)	24.8 ± 11.5
> 25 mm	130 (43.3%)
Knosp Grade	
0–2	215 (71.7%)
3–4	85 (28.3%)
Tumor Functional Type	
NF-PitNET	155 (51.7%)
GH-secreting PitNET	60 (20.0%)
PRL-secreting PitNET	45 (15.0%)
ACTH-secreting PitNET^a^	25 (8.3%)
TSH/Other	15 (5.0%)
Preoperative Hyponatremia (< 135mmol/L)	28 (9.3%)
Preoperative Pituitary Insufficiency^b^	130 (43.3%)
History of Tumor Apoplexy	20 (6.7%)
Recurrent Surgery	40 (13.3%)
Preoperative Fasting Time (hours), median (IQR)	10 (8–12)
Surgical Approach (Transsphenoidal)	275 (91.7%)
Operative Duration (minutes)	165 ± 55
> 180 min	95 (31.7%)

a. ACTH-secreting PitNET refers to patients with clinical Cushing’s disease

b. Pituitary Insufficiency refers to deficiency in at least one axis requiring replacement

### Incidence of early postoperative hyponatremia

EPH occurred in 70 (23.3%) patients. Regarding severity, the majority of EPH cases were mild (*n* = 52, 74.3%; 130–134 mmol/L), while 13 patients (18.6%) had moderate (125–129 mmol/L), and 5 (7.1%) presented with severe (< 125 mmol/L) hyponatremia.Univariate analysis showed that compared to the non-EPH group, the EPH group had a larger tumor diameter (*P* < 0.001), higher proportion of Knosp grade ≥ 3 (*P* < 0.001), higher proportion of ACTH-secreting PitNETs (*P* < 0.001), higher preoperative hyponatremia rate (*P* < 0.001), higher preoperative pituitary insufficiency rate (*P* = 0.035), higher recurrent surgery rate (*P* = 0.041), and longer operative duration (*P* < 0.001). Age, sex, history of tumor apoplexy, preoperative fasting time, and surgical approach showed no significant differences between the two groups (*P* > 0.10) (Table 2).

**Table 2:** Univariate analysis of early postoperative hyponatremia following PitNET surgery

Variable	EPH Group (*n* = 70)	Non-EPH Group (*n* = 230)	*P*-value
Age (years, Mean ± SD)	49.8 ± 11.5	48.1 ± 12.3	0.358
Sex (Female, %)	39 (55.7%)	121 (52.6%)	0.651
Max. Tumor Diameter > 25 mm (%)	45 (64.3%)	85 (37.0%)	< 0.001
Knosp Grade ≥ 3 (%)	38 (54.3%)	47 (20.4%)	< 0.001
Tumor Functional Type			
NF-PitNET (%)	30 (42.9%)	125 (54.3%)	0.095
GH-secreting PitNET (%)	12 (17.1%)	48 (20.9%)	0.501
PRL-secreting PitNET (%)	8 (11.4%)	37 (16.1%)	0.330
ACTH-secreting PitNET (%)	15 (21.4%)	10 (4.3%)	< 0.001
TSH/Other (%)	5 (7.1%)	10 (4.3%)	0.378
Preoperative Hyponatremia (%)	18 (25.7%)	10 (4.3%)	< 0.001
Preoperative Pituitary Insufficiency (%)	38 (54.3%)	92 (40.0%)	0.035
History of Tumor Apoplexy (%)	6 (8.6%)	14 (6.1%)	0.480
Recurrent Surgery (%)	14 (20.0%)	26 (11.3%)	0.041
Operative Duration > 180 min (%)	35 (50.0%)	60 (26.1%)	< 0.001

Transient diabetes insipidus requiring desmopressin treatment occurred in 35 patients. Importantly, only 4 (11.4%) of these patients subsequently developed EPH, suggesting that protocol-driven desmopressin use was not a major contributor to hyponatremia in this cohort.

### Multivariate logistic regression analysis

Multivariate binary logistic regression with stepwise selection identified five independent risk factors for EPH: tumor diameter > 25 mm, Knosp grade ≥ 3, ACTH-secreting PitNET, preoperative hyponatremia, and operative duration > 180 min. Preoperative pituitary insufficiency and recurrent surgery did not show independent predictive value (Table 3).

**Table 3:** Multivariate logistic regression analysis of independent risk factors for early postoperative hyponatremia

Independent Risk Factor	Regression Coefficient (β)	OR	95% CI	*P*-value
Max. Tumor Diameter > 25 mm	1.047	2.85	1.52–5.34	0.001
Knosp Grade ≥ 3	1.131	3.10	1.65–5.83	< 0.001
ACTH-secreting PitNET	1.267	3.55	1.41–8.94	0.007
Preoperative Hyponatremia	1.435	4.20	1.81–9.74	< 0.001
Operative Duration > 180 min	0.765	2.15	1.13–4.08	0.019
Constant	-3.556			< 0.001

To assess the robustness of the identified risk factors, a sensitivity analysis was performed by excluding the 28 patients with preoperative hyponatremia to eliminate potential confounding. In the remaining cohort of 272 patients, multivariate logistic regression confirmed that the identified predictors remained statistically significant: Maximum Tumor Diameter > 25 mm (OR = 2.91, 95% CI: 1.55–5.48, *P* = 0.002), Knosp Grade ≥ 3 (OR = 3.05, 95% CI: 1.61–5.75, *P* < 0.001), ACTH-secreting PitNET (OR = 3.62, 95% CI: 1.45–9.10, *P* = 0.008), and Operative Duration > 180 min (OR = 2.08, 95% CI: 1.09–3.98, *P* = 0.025) (Supplementary Table 1).

### Risk prediction model and scoring system

The logistic regression model was: Logit(P) = -3.556 + 1.047(Tumor diameter > 25 mm) + 1.131(Knosp grade ≥ 3) + 1.267(ACTH-secreting PitNET) + 1.435(Preoperative hyponatremia) + 0.765(Operative duration > 180 min).

**Fig. 1:**
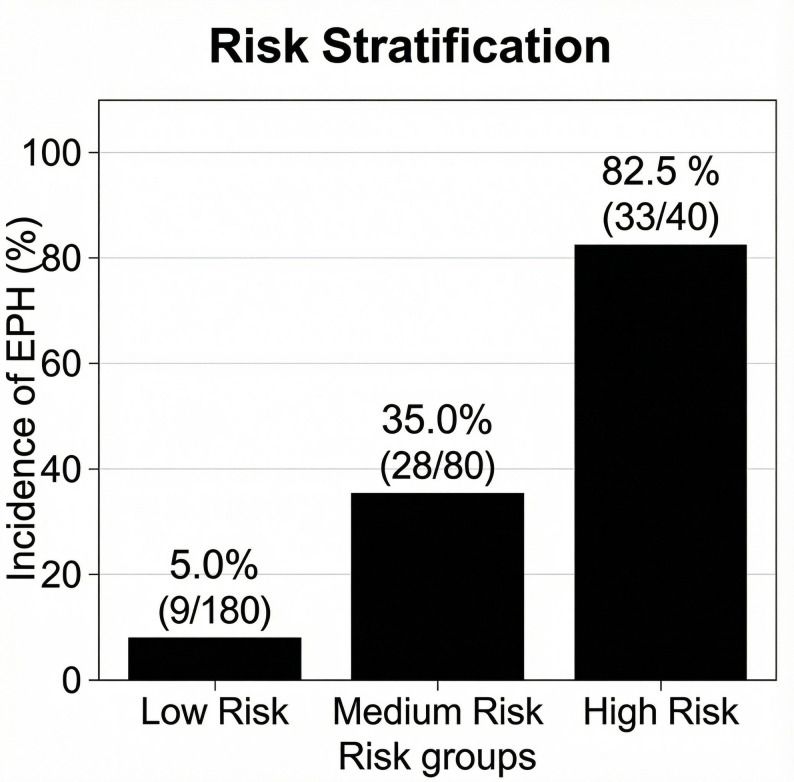
Incidence of early postoperative hyponatremia (EPH) by risk score category. The histogram displays EPH incidence (y-axis) across low- (0–1 points), medium- (2–3 points), and high-risk (≥ 4 points) groups (x-axis), with significant differences between groups (Pearson χ²=90.44, *P* < 0.001)

A simplified risk scoring system (0–6 points) was developed based on OR values (Table 4). Risk stratification showed significant differences in EPH incidence across groups: low-risk (0–1 points, 5.0%), medium-risk (2–3 points, 35.0%), and high-risk (≥ 4 points, 82.5%) (*P* < 0.001) (Table 5; Fig. 1). The optimal cutoff value of the risk score was 3 points, with a sensitivity of 78.6% and specificity of 83.9%.

**Table 4:** Risk scoring system for early postoperative hyponatremia following PitNET surgery

Risk Factor	Score
Max. Tumor Diameter > 25 mm	1
Knosp Grade ≥ 3	1
ACTH-secreting PitNET	1
Preoperative Hyponatremia	2
Operative Duration > 180 min	1
Total Score Range	0–6

**Table 5:** Comparison of EPH incidence across different risk score categories (*N* = 300)

Risk Score Category	Score Range	Total Patients (*N*)	EPH Cases (*n*)	EPH Incidence (%)
Low Risk	0–1	180	9	5.0
Medium Risk	2–3	80	28	35.0
High Risk	≥ 4	40	33	82.5
Total	0–6	300	70	23.3
Statistical Test Results				
Overall Comparison^a^				*P* < 0.001
Test for Trend^b^				*P* < 0.001

^a^ Pearson Chi-square test (χ²=90.44,*P* < 0.001); ᵇ Chi-square test for linear trend (χ²=84.45,*P* < 0.001)

### Model performance assessment

**Fig. 2:**
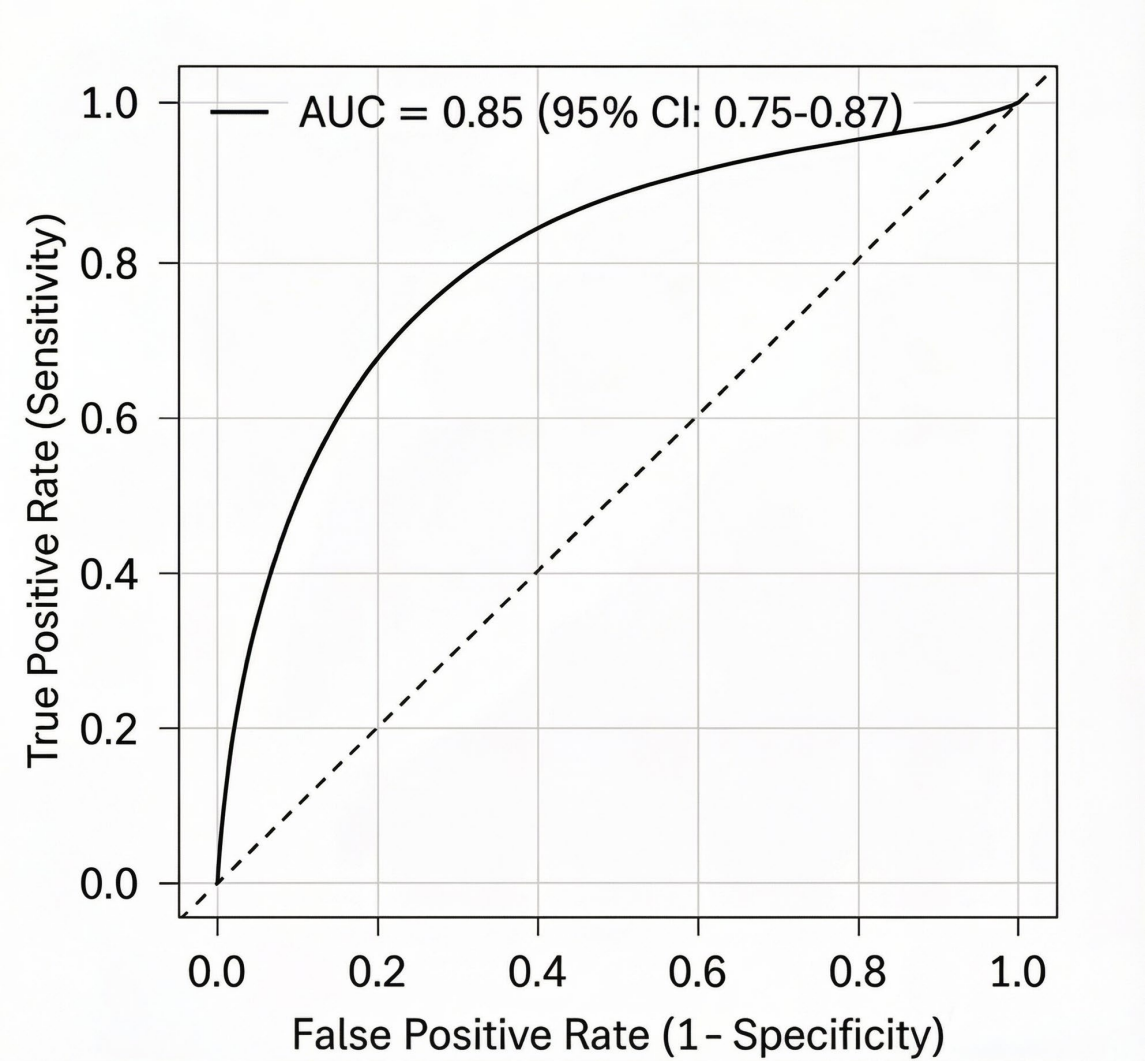
Receiver operating characteristic (ROC) curve for the EPH prediction model. The AUC is 0.85 (95% CI: 0.75–0.87), indicating excellent discriminative ability

**Fig. 3:**
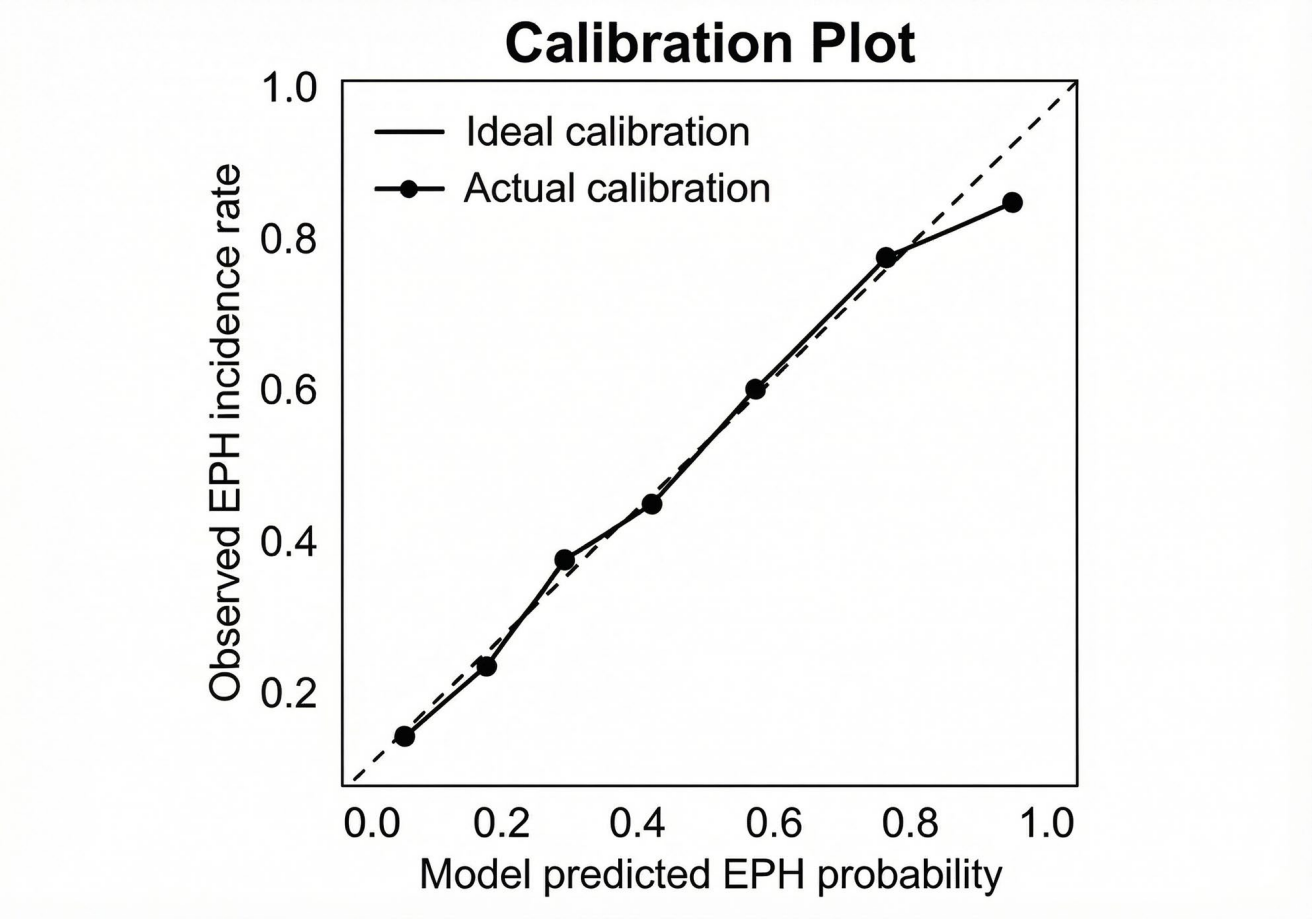
Calibration plot for the EPH prediction model. The solid line represents ideal calibration, and the dashed line represents actual calibration, showing good alignment between predicted probabilities and observed EPH frequencies

The predictive model exhibited excellent discriminative ability with an AUC of 0.85 (95% CI: 0.75–0.87)(Fig. 2). The calibration plot(Fig. 3) showed adequate agreement between the predicted and observed EPH probabilities, which was statistically confirmed by the Hosmer-Lemeshow test (χ²=8.56, df = 8, *P* = 0.38). Furthermore, internal validation using bootstrap resampling (1000 iterations) confirmed the robustness of the model, yielding an optimism-corrected AUC of 0.83.

## Discussion

This study systematically identified independent risk factors for EPH (POD 1–3) across all PitNET subtypes and developed a validated predictive model with a simplified scoring system. The key findings include a 23.3% incidence of EPH, five independent risk factors (tumor diameter > 25 mm, Knosp grade ≥ 3, ACTH-secreting PitNET, preoperative hyponatremia, operative duration > 180 min), and a high-performing predictive model (AUC = 0.85). These results address a critical gap in the literature, as most previous studies have focused on DPH (POD 4–10) or specific subtypes [8, 10], and provide novel insights into the unique pathophysiology and clinical management of EPH.

### Core innovations of the studycore innovations of the study

Three key innovations distinguish this work from existing literature:

#### Comprehensive subtype coverage

Unlike prior EPH studies limited to NF-PitNETs [10], our model covers all functional and nonfunctional PitNET subtypes, enabling its application to the entire PitNET surgery cohort. This addresses a major limitation of subtype-specific research and broadens the tool’s clinical utility.

#### Practical risk scoring system

We established a simplified 0–6 point scoring system with clear risk stratification (5.0–82.5% incidence) and an optimal cutoff (3 points) with high sensitivity (78.6%) and specificity (83.9%). The system uses five readily available variables, allowing rapid calculation by neurosurgeons, endocrinologists, and nurses without complex software—facilitating integration into electronic health record (EHR) decision-support tools.

#### Distinction between EPH and DPH

We systematically distinguished EPH from DPH by identifying tumor-specific (size, invasiveness, ACTH subtype) and perioperative (preoperative hyponatremia, operative duration) predictors, confirming EPH as a distinct clinical entity requiring targeted risk assessment.

### Pathophysiological mechanisms of independent risk factors

#### Tumor size and invasiveness (knosp grade)

Larger tumors (> 25 mm) and higher invasiveness (Knosp grade ≥ 3) were strong predictors of EPH, consistent with the notion that extensive tumor involvement of the hypothalamic-pituitary axis or cavernous sinus increases surgical trauma [11, 12]. Unlike DPH—where tumor size was not identified as a risk factor [8]—EPH is likely driven by acute structural damage to the pituitary stalk or hypothalamus during surgery for large, invasive tumors. This damage may disrupt the delicate balance of antidiuretic hormone (ADH) secretion or trigger a robust inflammatory response, leading to early sodium loss (similar to CSWS) or transient ADH dysregulation [9]. Additionally, larger tumors require more extensive surgical manipulation, increasing the risk of intraoperative injury to surrounding tissues and postoperative edema, which further exacerbates electrolyte disturbances.

#### ACTH-secreting PitNET

ACTH-secreting PitNETs (Cushing’s disease) are a unique risk factor for EPH, with an OR of 3.55. Preoperatively, patients with ACTH-secreting PitNETs exhibit chronic hypercortisolism, which inhibits ADH secretion [13, 14]. Consistent with recent findings [15], the abrupt decline in cortisol after tumor resection releases ADH inhibition, leading to early water retention. This mechanism is distinct from SIAD-related DPH [8] and highlights the need for tailored cortisol replacement in this subgroup. Notably, this risk factor was not identified in previous EPH or DPH studies [8, 10], underscoring the value of our study’s inclusion of all PitNET subtypes.

#### Preoperative hyponatremia

Preoperative hyponatremia was the strongest predictor of EPH (OR = 4.20), indicating that patients with preexisting water-sodium regulation impairment are more susceptible to decompensation following surgical stress. Preoperative hyponatremia likely reflects the patient’s status at admission due to mass effect-induced SIAD or secondary adrenal insufficiency. While these patients were optimized with hormone replacement prior to surgery, the persistence of risk postoperatively—despite universal steroid coverage—suggests a dominant role of SIAD mechanisms over simple hypocortisolism. This finding underscores the importance of preoperative assessment and correction of hyponatremia. While Zeng et al. [10] reported preoperative glucose elevation as a risk factor for EPH in NF-PitNETs, our study identifies preoperative hyponatremia as a more direct and clinically actionable predictor, as it reflects baseline electrolyte homeostasis rather than metabolic comorbidities.Importantly, our sensitivity analysis demonstrated that tumor-specific and surgical risk factors remain valid independent predictors even after excluding these patients, highlighting the robustness of the model.

#### Operative duration

Prolonged operative duration (> 180 min) was an independent risk factor (OR = 2.15), serving as a surrogate for surgical complexity and trauma. Longer surgeries are associated with increased intraoperative manipulation of the hypothalamic-pituitary region, higher blood loss, and greater fluid shifts—all of which contribute to early electrolyte imbalance. In contrast, Zeng et al. [10] focused on medication use (thiazide diuretics) and systemic factors (BMI, glucose) as EPH predictors, while our study emphasizes surgical and tumor-specific factors that are more directly modifiable through preoperative planning and intraoperative technique optimization.

### Comparison with Other EPH and DPH Studies

A key strength of this study is the direct comparison with recent EPH and DPH literature [8, 10]. Zeng et al. [10] recently reported an EPH incidence of 25.6% in NF-PitNETs, with independent predictors including thiazide diuretic use, pituitary apoplexy, preoperative glucose elevation, and BMI > 27 (protective factor). While our study’s EPH incidence (23.3%) is consistent with this finding, our work offers four critical advantages:

#### Broader patient coverage

Our model applies to all PitNET subtypes, whereas Zeng et al. [10] focused exclusively on NF-PitNETs.

#### Tumor-specific guidance

Our identification of tumor size, invasiveness, and ACTH subtype provides direct insights for preoperative surgical planning (e.g., resection strategy, expected duration), whereas Zeng et al. [10]’s predictors offer limited tumor-specific management guidance.

#### Practical scoring system

Our validated scoring system enables rapid bedside risk stratification, which Zeng et al. [10] did not provide.

#### Robust model validation

Our model includes bootstrap internal validation, AUC, and calibration assessments—unlike Zeng et al. [10], who did not report model calibration or quantitative predictive accuracy beyond OR values.

Compared to DPH studies [8], our findings further highlight the distinct nature of EPH: DPH is driven by intraoperative CSF leak, postoperative meningitis, and TSH elevation [8], while EPH is dominated by tumor characteristics, preoperative electrolyte status, and surgical duration. This distinction confirms that EPH and DPH are separate clinical entities requiring distinct risk assessment tools—with our study addressing the unmet need for an EPH-specific tool.

### Clinical translation of the predictive model and scoring system

The developed model and scoring system offer significant clinical value for risk-stratified perioperative management:

#### Preoperative risk stratification

The scoring system enables rapid identification of high-risk patients (≥ 4 points, 82.5% incidence), who may benefit from preoperative optimization (e.g., correction of hyponatremia, tumor downsizing) and intraoperative precautions (e.g., minimizing hypothalamic-pituitary manipulation).

#### Personalized postoperative monitoring

Low-risk patients (0–1 points, 5.0% incidence) can undergo standard monitoring (once daily); medium-risk patients (2–3 points, 35.0% incidence) require twice-daily serum sodium testing; high-risk patients (≥ 4 points) need intensified monitoring (every 6–8 h) to detect early hyponatremia.

#### Targeted Interventions

High-risk patients may benefit from isotonic/hypertonic saline supplementation (instead of hypotonic fluids) and strict intake-output monitoring. Nurses and physicians can use the risk score to prioritize resources and enhance interdisciplinary communication during handovers.

## Limitations and future directions

This study has several limitations. First, the single-center retrospective design may limit generalizability; future multicenter prospective studies are needed to validate the model in diverse patient populations and surgical settings. Second, detailed physiological data (e.g., urine osmolality, ADH levels) were not uniformly available, precluding definitive differentiation of EPH mechanisms (CSWS vs. early SIAD)—a gap that should be addressed in future mechanistic studies. Third, external validation was not performed; while bootstrap internal validation provides preliminary support, external cohorts are required for widespread clinical adoption. Finally, the impact of risk-stratified interventions on patient outcomes (e.g., EPH severity, hospital stay) was not evaluated, warranting prospective trials to confirm the model’s clinical benefit.

Future research should explore novel biomarkers for EPH prediction, such as copeptin (a surrogate for ADH activity) [4], which has shown promise in DPH studies [8]. Additionally, integrating machine learning algorithms with clinical data may further improve model performance by capturing complex interactions between risk factors.

## Conclusion

This study identifies five independent risk factors for early postoperative hyponatremia (EPH) following pituitary neuroendocrine tumor (PitNET) surgery: tumor diameter > 25 mm, Knosp grade ≥ 3, ACTH-secreting subtype, preoperative hyponatremia, and operative duration > 180 min. To our knowledge, this is an applicable predictive model and simplified scoring system for EPH across all PitNET subtypes. The validated model (AUC = 0.85) enables accurate preoperative risk stratification, facilitating personalized perioperative monitoring and targeted interventions to reduce EPH-related complications. This tool addresses a critical gap in clinical practice, improving patient safety and optimizing outcomes for PitNET patients undergoing surgery.

## Supplementary Information

Below is the link to the electronic supplementary material.

Supplementary Material 1

## Data Availability

The datasets generated and/or analyzed during the current study are available from the corresponding author on reasonable request.
