# Supplementary material for: Early postoperative hyponatremia after pituitary adenoma surgery: risk factors, predictive model, and clinical implications
Source: BMC Endocr Disord. 2026 Mar 3;26:99. doi: 10.1186/s12902-026-02212-2 (PMC13063525; doi:10.1186/s12902-026-02212-2)
Supplement: Supplementary file 1 — Supplementary Material 1 [file 12902_2026_2212_MOESM1_ESM.docx]

Supplementary Table 1. Sensitivity Analysis: Multivariate Logistic Regression for Early Postoperative Hyponatremia Excluding Patients with Preoperative Hyponatremia (n=272)

| Independent Risk Factor | Regression Coefficient (β) | Odds Ratio (OR) | 95% CI | P-value |
| --- | --- | --- | --- | --- |
| Max. Tumor Diameter > 25 mm | 1.068 | 2.91 | 1.55-5.48 | 0.002 |
| Knosp Grade ≥ 3 | 1.115 | 3.05 | 1.61-5.75 | <0.001 |
| ACTH-secreting PitNET | 1.286 | 3.62 | 1.45-9.10 | 0.008 |
| Operative Duration > 180 min | 0.732 | 2.08 | 1.09-3.98 | 0.025 |
| Constant | -3.812 | - | - | <0.001 |

This sensitivity analysis excluded 28 patients who presented with preoperative hyponatremia to eliminate potential confounding effects of baseline sodium status. The analysis confirms that tumor size, invasiveness, functional subtype (ACTH-secreting), and operative duration remain significant independent predictors of EPH in the eunatremic population.
